# A sliding window and Fourier decomposition method based 1D-CNN framework for motor imagery EEG classification

**DOI:** 10.3389/fncom.2026.1945160

**Published:** 2026-10-02

**Authors:** B. S. S. Tejesh, Karra Bhaskar, M. Sivaji, Ravisankar Dakupati, Saswat Kumar Ram, Pradyut Kumar Sanki, M. Ramakrishnan

**Affiliations:** Department of Electronics and Communication Engineering, SRM University-AP, Amaravati, Andhra Pradesh, India

**Keywords:** brain computer interface, convolutional neural network, electroencephalography, Fourier decomposition method, Jetson Nano, motor imagery, sliding window

## Abstract

Electroencephalography (EEG) signal classification is fundamental requirement for effective motor imagery (MI) brain-computer interface (BCI) applications. However, current MI classification methods often depend on handcrafted spatial filtering or complex time-frequency transformations that increase computational complexity. To mitigate this limitation, we propose a unified architecture that integrates a sliding window (SW), a Fourier decomposition method (FDM), and a one-dimensional convolutional neural network (1D-CNN) for MI-EEG classification. The raw EEG data were segmented using the SW approach to augment the data and extract more temporal feature patterns from each trial. The segmented signal, containing time-domain features, was subsequently processed using a band-pass filter and FDM to extract frequency-based features. Here, FDM decomposes the signal into eight orthogonal Fourier Intrinsic Band Functions (FIBFs). A 1D-CNN architecture was employed to learn representations from the decomposed EEG signals. The proposed framework was evaluated on the BCI Competition III Dataset IVa (BCIC III IVa) and BCI Competition IV-2a (BCIC IV 2a) datasets. Our method achieved accuracies of 79.14% and 93.67% on the BCIC IV 2a and BCIC III IVa datasets, respectively, under the subject-dependent evaluation protocol. To evaluate cross-subject generalization, the proposed framework was validated using the Leave-One-Subject-Out (LOSO) protocol, achieving average classification accuracies of 76.49% on the BCIC III Dataset IVa and 55.50% on the BCIC IV 2a dataset. Furthermore, deployment on the NVIDIA Jetson Nano demonstrated complete pipeline latencies of 58.119 ms/trial and 85.055 ms/trial, corresponding to throughputs of 17.21 and 11.76 trials/s for the BCIC III IVa and BCIC IV 2a datasets, respectively. The results show the strong potential of the developed MI-EEG classification method to assist subjects with motor ailments.

## 1 Introduction

Neurotechnology, termed a BCI, enables a link between the user's brain and a computing system (Edelman et al., 2024). With the aid of brain signals, subjects can communicate with devices using commands (He et al., 2020). Patients with motor ailments can communicate their requirements using BCI. BCI technology assists patients with amyotrophic lateral sclerosis (ALS) (Sellers and Donchin, 2006), stroke, and spinal cord injury (Müller-Putz et al., 2014) in performing their regular activities. Moreover, BCI can be divided into invasive and noninvasive types (Zhang et al., 2023; Zhou et al., 2019). Invasive BCIs ensure highly detailed brain information, high-quality brain activity recordings, and high safety considerations for the subject; however, their use is not recommended for all subjects due to safety considerations. In contrast, noninvasive BCIs can be easily adopted without high safety measures, and brain activity can be recorded for the duration of the event.

EEG is of predominant importance due to its widespread use in research and medical applications. EEG is a noninvasive method that adheres electrodes to the scalp to measure the electrical activity for studying and examining brain signals (Sharma et al., 2023a). EEG electrodes measure voltage fluctuations induced by ionic activity in the brain. Motor-related brain activity involving both voluntary and imagined movements reflects changes in brain signals, such as sensorimotor rhythms (SMRs). Among the various signals recorded from the brain, MI signals are elicited when a subject imagines performing a motor task while remaining physically motionless. An artificial limb, wheelchair, or any external device can be triggered based on MI signals associated with specific body parts.

To enhance classification accuracy, several challenges, such as low signal-to-noise ratio (SNR), artifacts from electronic devices, a low information transfer rate, eye blinking, muscle movements, high dimensionality and subject dependency degrade EEG signals (Altaheri et al., 2022). A BCI framework comprises crucial steps, including signal acquisition, preprocessing, feature extraction, and classification. When EEG signals are captured, different kinds of noise and artifacts may disturb the recordings such as movements, eye blinking, and other environmental interference. Thus, preprocessing methods need to be utilized, including filtering, artifact removal, and denoising methods. Subsequently, discriminative features are extracted and provided to a classifier for accurate motor MI recognition. Despite these advancements, reliable decoding of MI-EEG signals remains a major bottleneck for BCI systems.

Modern MI-EEG classification methods deteriorate owing to the subsequent constraints. First, most MI-EEG analyses do not capture short temporal patterns, which may result in inadequate feature detection (Ma et al., 2023). Second, frequency analysis techniques employ fixed filter banks that may fail to capture the dynamic neural activity (Nieto et al., 2021). Third, high-dimensional features are extracted, leading to overfitting and reduced classifier performance (Zhou et al., 2023). Finally, many pipelines lack a unified structure for both temporal segmentation and feature compression, which significantly slows down real-time processing. These constraints indicate the importance of a more efficient and generalizable approach to MI-EEG classification.

In this paper, temporal feature extraction and data augmentation were performed using the sliding window method from raw EEG recordings. Each segmented window obtained through the sliding window method was decomposed using a butterworth band-pass filter and the FDM seperating the signal into FIBFs to capture the multifrequency characteristics. The decomposed FIBFs were provided to the 1D-CNN for automatic feature extraction in MI classification.

The key advancements presented in this paper are listed below.

1. We propose a unified trial-level temporal-spectral MI-EEG classification framework for robust motor imagery decoding that combines temporal and spectral information to improve classification performance.
2. The proposed approach employs FDM-based spectral representation for MI-EEG signal to decompose EEG signals into FIBFs, thereby enabling intrinsic band-level characterization. The decomposed FIBFs are provided to the 1D-CNN to automatically obtain features without relying on handcrafted features.
3. An efficient lightweight 1D-CNN architecture is employed to obtain temporal and spectral features extracted from the FDM-decomposed signals while maintaining low computational complexity suitable for embedded platforms.
4. The proposed model was assessed under subject-dependent and subject-independent LOSO protocols on the BCIC III IVa as well as BCIC IV2a datasets to assess cross-subject generalization.
5. An inference evaluation on the Jetson Nano platform is conducted using the BCIC III IVa and BCIC IV 2a datasets to analyze the performance of the developed framework.
6. A systematic ablation study is performed to quantitatively analyze the individual contributions of the Sliding Window (SW) segmentation, FDM-based intrinsic band decomposition, and 1D-CNN-based representation, thereby assessing the effectiveness of each module within the proposed framework.

## 2 Literature review

Attallah et al. (2020) proposed a hybrid feature extraction framework by integrating wavelet packet decomposition and time-domain features on the BCIC III IVa dataset. The method was evaluated using different channel subsets of 3, 18, and 25 channels. The 18-channel subset covered the central (sensorimotor) and parietal regions. Cross-subject generalization was not evaluated. Ramoser et al. (2000) implemented a method termed Common Spatial Patterns (CSP) to design spatial filters, where the variances are optimized for the discrimination of MI tasks. When considering the same class of spatial features, the variance is minimized; however, for different classes, it is maximized. Similarly, Wang et al. (2006) stated that CSP employs spatial filters by performing a diagonalization operation on class-specific covariance matrices. Dornhege et al. (2005) stated that the Common Spatial-Spectral Pattern (CSSP) employed a frequency filter for each channel along with spatial filters.

Quadrianto et al. (2007) developed an improvement for CSP referred to as sub-band CSP (SBCSP), where EEG signals are divided into sub-bands with the aid of filter banks, and discriminative analysis is used for feature extraction, whereas the scores of SBCSP features for each band are employed for classification using Linear Discriminant Analysis (LDA). Ang et al. (2008) overcame the issue of CSP, which requires manual selection of the frequency range, by introducing Filter Bank CSP (FBCSP) to automatically select temporal-spatial EEG features implemented in four stages: frequency filtering, spatial filtering, feature selection, and classification. Thomas et al. (2009) introduced Discriminative FBCSP (DFCSP) to extract subject-dependent features from a set of filter banks. The subject-specific frequency components are acquired using the parent filter bank and then provided for CSP processing.

Huang and Wei (2024) employed Regularized CSP (RCSP) to handle CSP issues, such as noise and overfitting, in small EEG datasets by introducing regularization by incorporating penalty terms during computation by regularizing covariance matrices. Goksu et al. (2011) used sparse CSP (SCSP) for extracting features only from a subset of channels. By utilizing SCSP, the computational complexity was reduced using a greedy search-based generalized eigenvalue decomposition. Here, during optimization, a sparsity-inducing penalty was added, resulting in implicit channel selection and making some filter values zero. Asadi et al. (2025) discussed the Kernel CSP (KCSP), which is capable of capturing nonlinear relationships. The KCSP functions on covariance matrices employing a kernel function to assess similarities and transform the data into higher-dimensional feature space. Shi et al. (2024) stated that the spatial covariance matrix plays a predominant role in classification; however, its computation is intensive because it involves calculating distances between matrices and performing eigenvalue decomposition. In the Riemannian approach, the features of EEG are considered from the covariance matrices, and subsequently, processing is performed on Riemannian manifolds.

Maji and Pal (2016) detailed the evaluation of two adaptive signal decomposition methods, namely, Empirical Mode Decomposition (EMD) and Variational Mode Decomposition (VMD). EMD resolves the signal into multiple Intrinsic Mode Functions (IMFs), which indicate oscillatory components from the signal. VMD in an adaptive manner decomposes a signal into multiple band-limited IMFs, enabling the efficient separation of the frequency components. EMD is faster but limited in terms of the separation of signal components, whereas VMD is robust to noise and can preserve the signal components. VMD is more computationally intensive than EMD. Singh et al. (2017) proposed the FDM, which relies on Fourier theory for the evaluation of non-stationary signals and decomposes the input signal data into a set of FIBFs. It presents a flexible Fourier expansion of a time series represented by various amplitudes and frequencies generated using the Fourier transform. It has various properties, such as adaptability, implying that it is tailored to the specific signal; orthogonality, assuring non-redundancy as well as energy preservation in signal components; and zero mean, signifying that each FIBF has a mean of zero and represents only oscillatory components across the signal. Sadiq et al. (2022) implemented Multivariate VMD (MVMD) for multichannel EEG data; by utilizing it, features can be extracted by IMFs with statistical measures. It preserves the target information and eliminates incorrect data decomposition. Kevric and Subasi (2017) contributed to the comprehensive assessment of three distinguished signal decomposition methods, such as discrete wavelet transforms (DWT), empirical mode decomposition (EMD), and wavelet packet decomposition (WPD), for the MI-EEG classification task.

Hernández-González et al. (2021) used continuous wavelet transform (CWT) to acquire time-frequency images referred to as scalograms, and it provides significant multi-resolution analysis, which enables effective localization for high and low frequencies. It can precisely reconstruct and deconstruct signals. It is feasible to identify artifacts in data. Prakash (2018) illustrated that the WT has been adopted with several mother wavelets, such as Haar wavelets, which are used to detect unexpected variations in the signal, and the Shannon wavelet is equipped with adept frequency localization. Kant et al. (2020) implemented CWT along with Deep Learning (DL) based transfer learning. However, it suffers from discontinuity, aliasing, and oscillations in transformed signals. Wang Z. et al. (2018) validated that a CNN trained on STFT features contributes the best MI classification over established DL models. Muna et al. (2025) claimed that transformers, when ensembled with STFT, capture spatial, temporal, and spectral features. However, it suffers from a resolution problem in which wide windows exhibit reduced time resolution, whereas narrow windows exhibit poor frequency resolution. Al-Saegh (2023) systematically compared STFT, CWT, MODWT, and MODWTMRA for MI-EEG classification and a shallow CNN classifier. The study showed that the selection of signal-processing representation has a direct influence on classification performance. In particular, maximal-overlap wavelet methods, including Maximal Overlap Discrete Wavelet Transform, Maximal Overlap Discrete Wavelet Transform Multiresolution analysis, achieved better performance than STFT and CWT, with Symlets providing the strongest results. The study also highlighted the time–frequency resolution trade-off associated with STFT, where shorter windows improve temporal resolution at the expense of frequency resolution, whereas longer windows improve frequency resolution but reduce temporal resolution.

Belwafi et al. (2018) introduced an embedded BCI, which accomplishes the analysis of adaptive filtering while assessing event-related synchronization/desynchronization. LDA was employed to classify the obtained signals. Zhang et al. (2019) developed a hybrid DL model, which comprises two segments. In the first segment, preprocessing and feature extraction were performed using a one-vs.-rest filter bank. In the second segment, CNN and Long Short-Term Memory (LSTM) are utilized to extract hierarchical feature representations. Dai et al. (2020) used a hybrid scale CNN model, which is capable of extracting spatial as well as temporal features. The CSP is used as a spatial-filtering technique. Dokur and Olmez (2021) proposed a CNN model with decreased model complexity due to a reduction in the number of hyperparameters. Rashid et al. (2021) presented a random subspace K-Nearest Neighbors (kNN), which is an ensemble classifier. They trained random subsets on multiple kNN classifiers built upon the features extracted using CSP and features selected using Random Forest. Sakhavi et al. (2018) presented a pipeline where FBCSP is employed for spatial filtering and selection of features, extraction of temporal patterns from EEG signals using HT, and the extracted features are learned using a three-layered CNN. Lu et al. (2019) implemented a hybrid model with a composition of a sliding window along with 1D CNN and LSTM. The model was adopted to learn temporal information in the signal. The 1D CNN captures local temporal patterns, whereas the LSTM is used to extract long-term dependencies in the time series. Yang et al. (2015) put forward a method where Augmented CSP (ACSP) is used to extract features across overlapping frequency bands for capturing additional information; the ACSP features are tabulated in the form of a feature map and given to a CNN to learn discriminative features. Qiao and Bi (2019) presented a hybrid model that constitutes a CNN and a Bidirectional Gated Recurrent Unit (BGRU). The BGRU was adopted to acquire the relevant information extracted using a CNN. Riyad et al. (2021) developed EEGNET, which is inspired by ConvNet based on the architectures of Inception and Xception. To reduce the computational cost and lower the model parameters, separable and depth-wise convolutions were employed to extract effective temporal and spatial features, and parallel convolutions were implemented using an inception block. Zhao et al. (2019) presented a deep CNN (ConvNet) that aims to extract features from combined spatio-temporal-frequency patterns. In this architecture, the first convolutional layer extracts time-frequency patterns employing Morlet wavelet kernels over convolutional kernels to lower the parameter count and decrease the risk of overfitting, and the consequent layers accomplish spatial filtering. Liao et al. (2020) implemented three models using Shallow ConvNet. The first model uses multiple local spatial convolutional layers, the second model uses single global spatial convolution, and the third model acts as a parallel architecture that incorporates features from both local and global spatial convolution. Wang P. et al. (2018) implemented MI classification by employing LSTM. An approximation technique named one-dimension aggregate approximation (1d-AX) is utilized employed to capture salient signal features. The 1d-AX ensures noise suppression and dimensionality reduction, enabling the LSTM to consider significant temporal dependencies. Zhang et al. (2021) proposed a framework that associates a hybrid deep neural network (HDNN) with transfer learning. The HDNN internally comprises a CNN and LSTM to extract the temporal and spatial features. Li et al. (2022) employed CNN and LSTM with parallel connection. Spatial features are extracted by CNN and temporal features by LSTM. Sharma et al. (2023b) implemented FDM-based decomposition and captured statistical feature matrices and measured their performance using ML algorithms. Saideepthi et al. (2023) implemented the EEGNet model along with a sliding window and used the longest consecutive repetition as a post-processing step. Al-Saegh et al. (2024) investigated computationally efficient MI-EEG classification using a DCNN by combining EEG data reduction and network quantization. Data reduction was performed through signal downsampling and reduction of the number of EEG electrodes, while the trained network parameters were quantized from 32-bit floating-point to lower-precision fixed-point representations. The study reported that downsampling could reduce training time by more than 28% with only a small reduction in classification accuracy, while 8-bit fixed-point quantization reduced memory requirements and provided approximately a 2.7 × inference speedup. These findings demonstrate the importance of reducing both signal-level and model-level computational complexity for practical MI-EEG BCI deployment.

Han et al. (2025) proposed spatial-spectral and temporal dual prototype network for MI BCI, which integrates adaptive spatial-spectral fusion, multi-scale variance pooling, and dual prototype learning to extract spatial-spectral relationships, long-term temporal information, and discriminative class representations. They stated that their framework requires the incorporation of prior knowledge such as electrode mirror distribution and functional brain partitioning, the effectiveness of the dual-prototype network remains largely unexplored. Mai et al. (2024) introduced a dual stream neural network that jointly extracts spatial, spectral and temporal information from MI-EEG signals. To represent the spatial distribution of scalp activity, they transformed the EEG signals into temporal and spectral topographic map sequences. In both streams, CNN are employed to extract spatial features, BiLSTMs capture spectral dynamics across frequency frames and temporal dynamics across time frames. The representations obtained by the two streams are thereafter integrated through an attention-based neural network that selectively weights the most important temporal and spectral components before classification. They acknowledge that projecting three dimensional EEG distribution onto two dimensional topographic maps may lead to distortion and three dimensional modeling can increase the computational complexity. Zhi et al. (2024) proposed a Domain Generalization Framework with Supervised Contrastive Learning for Cross-Subject Motor Decoding, they employed multiscale spatial and temporal convolutions to extract discriminative EEG features, Deep CORAL based domain alignment reduces distribution differences among subjects. The method integrates supervised contrastive learning with domain-agnostic MixUp to strengthen class-level alignment and enhance generalization to unseen subjects. They acknowledge that the method still relies on domain labels for alignment and has not been sufficiently validated in cross-dataset and cross-task settings. Song et al. (2025) proposed a latent-distribution domain generalization framework for calibration-free cross-subject MI-EEG classification. Multiple EEGNet and shallowConvNet based branches are employed to capture diverse latent source-domain distributions, while a score network assesses the reliability of each branch and a gating mechanism adaptively fuses their predictions for decoding unseen subjects. The authors acknowledge that source-subject EEG variability is not explicitly considered, as low-SNR and less discriminative signals may adversely affect the training process.

Overall, existing MI-EEG approaches range from handcrafted spatial and spectral feature methods to decomposition based techniques, hybrid deep networks, and recent attention and domain generalization based architectures (Ramoser et al., 2000; Ang et al., 2008; Sharma et al., 2023b; Lu et al., 2019; Muna et al., 2025; Han et al., 2025; Zhi et al., 2024; Song et al., 2025). In comparison, the proposed framework adopts a unified temporal–spectral strategy in which sliding-window segmentation captures localized temporal variations, FDM decomposes each window into frequency-specific FIBFs, and the resulting components are directly provided to a lightweight 1D-CNN for automatic feature learning. Furthermore, unlike sliding-window approaches combined with CNN–LSTM or EEGNet (Lu et al., 2019; Saideepthi et al., 2023), the proposed framework integrates localized temporal segmentation and FDM-based spectral decomposition within a single lightweight 1D-CNN pipeline. Compared with recent transformer, attention, multi-branch, and domaingeneralization-based methods, the proposed framework uses a comparatively lightweight architecture while jointly exploiting localized temporal and frequency-specific information. Thus, the novelty lies in the complementary integration of SW, FDM, and lightweight 1D-CNN-based feature learning rather than in the individual use of these components.

## 3 Materials and methodology

The architecture of the developed system for the BCIC III IVa and BCIC IV 2a datasets using Sliding Window, FDM, and 1D-CNN is depicted in Figure 1. The proposed framework was evaluated for both MI-EEG binary classification involving right-hand and foot imagery classes and multiclass classification involving right-hand, left-hand, tongue, and both-feet MI classes.

**Figure 1:**
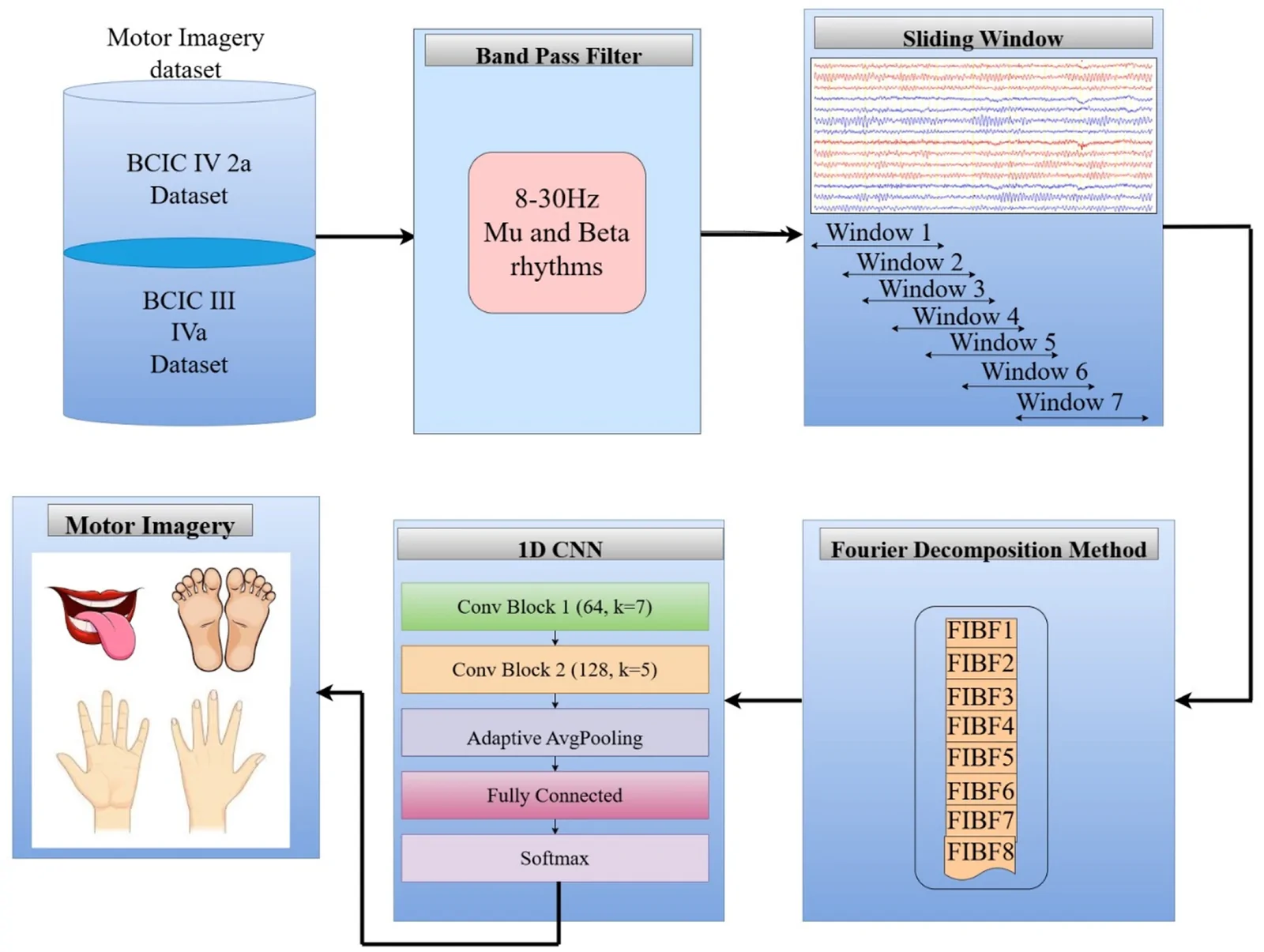
Proposed framework for MI-EEG classification.

### 3.1 Dataset

In this work, to enhance the classification efficiency, we chose the BCIC III IVa dataset for binary classification and BCIC IV 2a dataset for multi-class classification. The standard 10-20 system was employed for the datasets. The timing structure for the BCIC III IVa and BCIC IV 2a experimental paradigms is depicted in Figures 2, 3. The BCIC III IVa dataset comprises five individuals and two MI tasks, such as the right hand and foot. The signals were sampled at 100 Hz. Visual cues were displayed for 3.5 s to all five individuals. The number of original trials per subject was 288. Following the dataset protocol, trials annotated as containing artifacts were excluded from further analysis. Furthermore, the EOG channels included in the dataset were omitted from the EEG analysis. The channels that are proximate to MI are selected; therefore, instead of 118 channels, we chose only 22 channels. The selected EEG channels were located over the frontal, fronto-central, central, centro-parietal, and parietal areas, which encompass the sensorimotor cortex and are known to capture MI-related neural activity. The BCIC IV 2a dataset comprises data from nine individuals and four MI tasks, including tongue, left-hand, right-hand, and foot movements. Each recording session contained 288 trials per subject. The signals were sampled at 250 Hz. The visual cues were initiated at 2 s, and the MI task was completed at 6 s. Data from both recording sessions were combined and stratified at the trial level into 80% training and 20% testing sets. A fixed random seed of 42 was used for the main experiments to ensure reproducibility to deploy on Jetson Nano, while additional seeds of 512 and 1,023 were tested to check the consistency of the model performance. The model trained with seed 42 was subsequently deployed on the Jetson Nano for offline evaluation.

**Figure 2:**
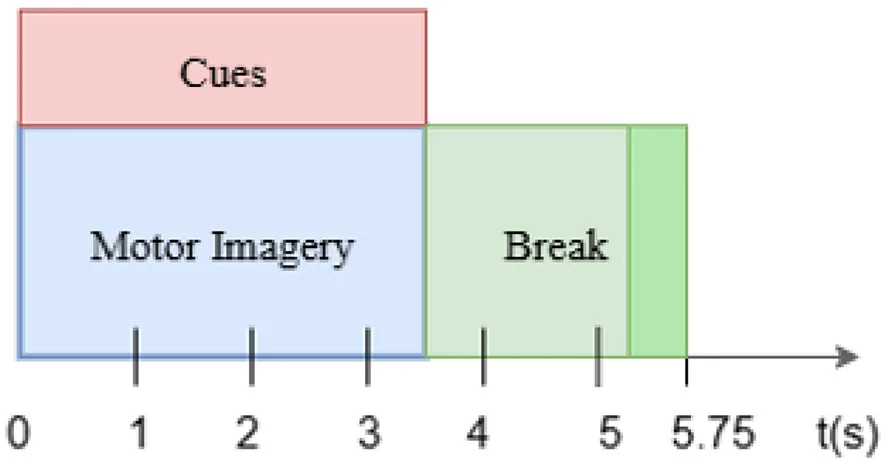
Timing structure of the BCIC III IVa experimental paradigm.

**Figure 3:**
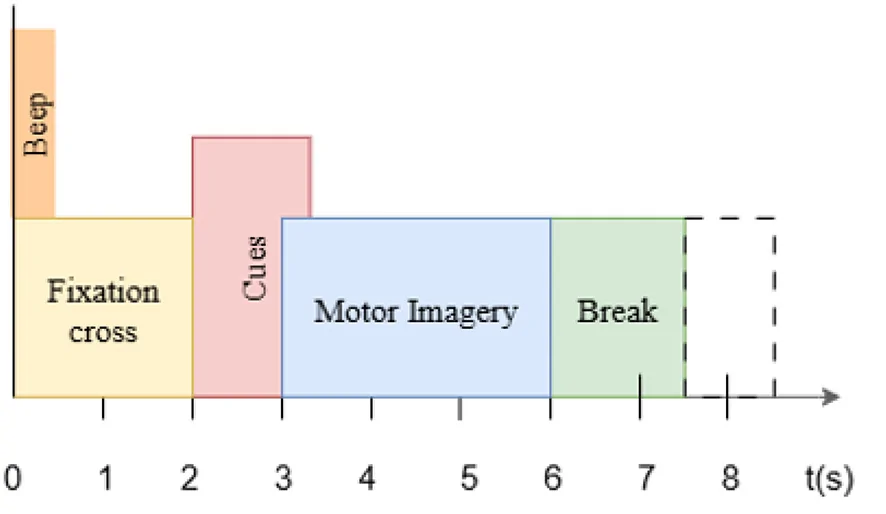
Timing structure of the BCIC IV 2a experimental paradigm.

### 3.2 Sliding window technique

A data augmentation technique termed SW was employed to improve classification accuracy for MI-EEG data. The temporal segmentation of single-trial EEG data employing sliding windows is shown in Figure 4. This approach was adopted for the analysis to segment the time-series data and ensure that effective temporal features are captured. By using overlapping segments in the SW, more training samples are generated, and the model learns better transitions. This ensures that no pattern in the signal is skipped. It also converts variable-length signals into fixed-size segments, making them compatible with ML models. From an analytical perspective, we considered the total trial length as *t* (samples), for sliding across the data window size *w* and step size considered as *s*. The number of windows *N* formed depends on *t*, *w*, and *s*, as shown in Equation 1. The SW approach with seven windows per trial was chosen because it ensured coverage of the complete MI event. Fewer than seven windows may miss temporal patterns, whereas more than seven windows increase unnecessary overlap (Singh et al., 2024). This tactic resulted in a 7 × increase in the dataset size, significantly strengthening the learning capacity of the model. The matrix is shaped into *t* × *C*, where *C* signifies the channels count. Here the MI trial expressed as a matrix *X* ∈ ℝ^*t*×*C*^, each trial is referred to as *X*_*i*_ and associated label as *y*_*i*_ ∈ {1, …, *K*}, the number of MI classes is represented with *K*. The labeled data ${\left\{D=\left\{\left(X^{\left(i\right)},y^{\left(i\right)}\right)\right\}\right\}}_{i=1}^{N}$, where *X*_*i*_ denotes the EEG trial and *y*_*i*_ represents its class label. *N* denotes the number of trials. For the BCIC III IVa dataset the *t* = 350, *w* = 230, *s* = 20 so total number of windows is 7, *K* = 2 as it is a binary classification and the timeline *t*_*w*_ for each window is 2.3 s with a shift *t*_*s*_ of 0.2 s and *t*_*rem*_ of 1.05 s in Equation 2, For the BCIC IV 2a dataset *t* = 1000, *w* = 700, *s* = 50, *K* = 4 as it is a multiclass classification and the timeline *t*_*w*_ for each window is 2.8 s with a shift *t*_*s*_ of 0.2 s and *t*_*rem*_ of 1.2 s, was chosen to ensure that each segment extracts the full MI event. The resulting segmented windows serve as inputs to the bandpass filter and FDM, preserving temporal patterns and improving generalization across binary and multiclass classification.


$$\begin{array}{l} N=\left[\left(\frac{t-w}{s}\right)\right]+1 \end{array}$$ (1)

$$\begin{array}{l} t_{rem}=\frac{t}{f_{s}}-t_{w} \end{array}$$ (2)

**Figure 4:**
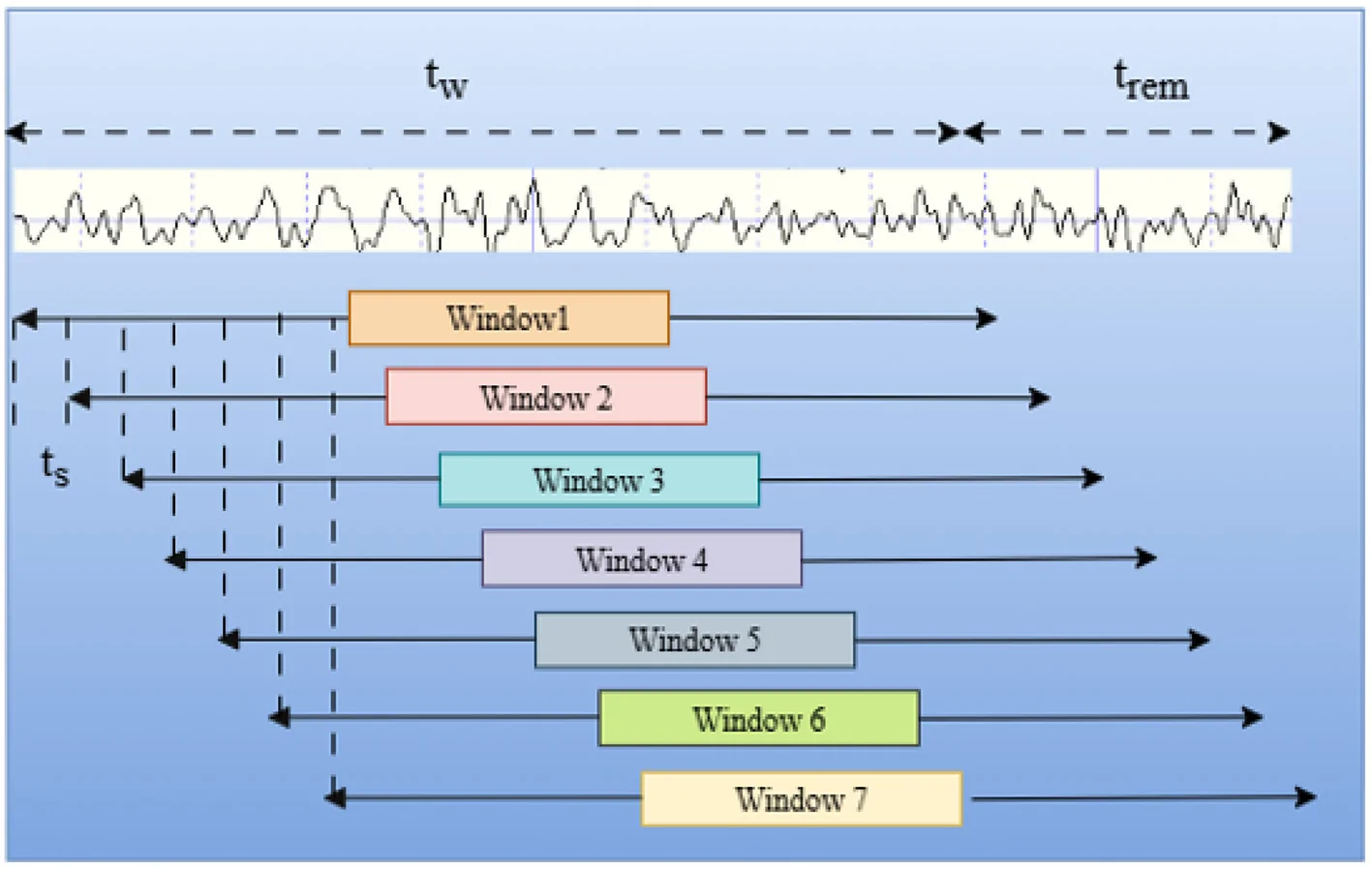
Temporal segmentation of single-trial EEG data using sliding windows.

### 3.3 Fourier decomposition method for EEG-MI processing

FDM is a powerful signal processing approach in which the signal is divided into a group of zero-mean components referred to as FIBFs, each of which comprises a specific frequency band with relevant cut-off frequencies. The FIBFs are intrinsic to the signal itself, the original signal can be precisely regenerated, local time-varying features are captured, they are data-driven, and redundancy among them is minimized (Sharma et al., 2022). Based on the signal frequency content, it is effective for analyzing nonstationary signals.

EEG signals typically exhibit low amplitude, high sensitivity to noise, and wideband frequency distribution. A bandpass filter was employed to enhance the signal quality by retaining only oscillations specific to MI. By applying a bandpass filter before the FDM, unwanted components or non-informative sub-bands are filtered out. This restricts the signal to the MI frequency range.

The signal was sampled at *f*_*s*_, where *f*_*s*_ is 250 Hz for multiclass classifications and *f*_*s*_ is 100 Hz for binary classification. The Nyquist frequency, *f*_*nyq*_ is defined as Equation 3 and the normalized filter cut-off frequencies ω_*low*_ and ω_*high*_ in Equations 4, 5 are calculated using *f*_*low*_ = 8 Hz and *f*_*high*_ = 30 Hz.


$$\begin{array}{l} f_{nyq}=\frac{f_{s}}{2} \end{array}$$ (3)

$$\begin{array}{l} \omega_{low}=\frac{f_{low}}{f_{nyq}} \end{array}$$ (4)

$$\begin{array}{l} w_{high}=\frac{f_{high}}{f_{nyq}} \end{array}$$ (5)

The bandpass filter is designed with the transfer function *H*(*z*) in Equation 6, which specifies how each frequency component is scaled and phase shifted. The time-domain implementation of the filter is illustrated using a difference equation, resulting in the filtered output *y*[*n*] in Equation 7.


$$\begin{array}{l} H\left(z\right)=\frac{\sum_{k=0}^{N}b_{k}z^{-k}}{1+\sum_{k=1}^{N}a_{k}z^{-k}} \end{array}$$ (6)

$$\begin{array}{l} y\left[n\right]=\overset{N}{\underset{k=0}{\sum }}b_{k}x\left[n-k\right]-\overset{N}{\underset{k=1}{\sum }}a_{k}y\left[n-k\right] \end{array}$$ (7)

The filtered signal *y*[*n*] comprises solely the frequency components of the MI-EEG, which serves as the input to the Fast Fourier Transform (FFT)-based FDM, which divides the spectrum into multiple sub-bands. The FFT is represented using Equation 8, and the inverse FFT (IFFT) reconstructs the signal from its frequency spectrum using Equation 9. Here, *L* denotes the signal length.


$$\begin{array}{l} E\left[k\right]=\overset{L-1}{\underset{n=0}{\sum }}y\left[n\right]e^{-j\frac{2\pi kn}{L}} \end{array}$$ (8)

$$\begin{array}{l} y\left[n\right]=\frac{1}{L}\overset{L-1}{\underset{k=0}{\sum }}E\left[k\right]e^{j\frac{2\pi kn}{L}} \end{array}$$ (9)

Δ*f* is the frequency spacing between the FFT bins represented by Equation 10, where each bin corresponds to a particular frequency in hertz. The term *k* corresponds to the index of a discrete frequency bin in the FFT, as expressed in Equation 11.


$$\begin{array}{l} \Delta f=\frac{f_{s}}{L} \end{array}$$ (10)

$$\begin{array}{l} f\left[k\right]=k\Delta f \end{array}$$ (11)

The *k*_*low*_ and *k*_*high*_ in Equation 12 indicate the start and end points of the band inside the FFT index axis, respectively. The range between *k*_*low*_ and *k*_*high*_ represents the frequency band of interest.


$$\begin{array}{l} k_{low}=\frac{f_{low}}{\Delta f},\;k_{high}=\frac{f_{high}}{\Delta f} \end{array}$$ (12)

Equation 13 splits the MI-EEG frequency band into *n*_*fibf*_ equal subbands, indexed as *i* = 0, 1, …, *n*_*fibf*_.


$$\begin{array}{l} k_{i}=k_{low}+i\left(\frac{k_{high}-k_{low}}{n_{fibf}}\right) \end{array}$$ (13)

The band-pass signal *y*[*n*] in Equation 14 can be represented as the sum of its FIBFs. Each FIBF is denoted by *A*_*i*_[*n*] and refers to a specific sub-band within the overall MI-EEG range.


$$\begin{array}{l} y\left[n\right]=\overset{n_{fibf}}{\underset{i=1}{\sum }}A_{i}\left[n\right] \end{array}$$ (14)

The binary mask function *p*_*i*_[*k*] in Equation 15 is used for subband selection in the frequency domain, which is specified as 1 for FFT bins within the *i*^*th*^ subband and 0 otherwise.


$$\begin{array}{l} p_{i}\left[k\right]=\{\begin{array}{ll} 1, & k_{i}\leq k<k_{i+1}\;\text{and}\;L-k_{i+1}\leq k<L-k_{i}\\ 0, & \text{otherwise} \end{array} \end{array}$$ (15)

The frequency components corresponding to the subbands are preserved by multiplying the full spectrum *E*[*k*] with the binary mask *p*_*i*_[*k*] as in Equation 16.


$$\begin{array}{l} E_{i}\left[k\right]=E\left[k\right]\cdot p_{i}\left[k\right] \end{array}$$ (16)

$$\begin{array}{l} A_{i}\left[n\right]=\frac{1}{L}\overset{L-1}{\underset{k=0}{\sum }}E_{i}\left[k\right]e^{j\frac{2\pi kn}{L}} \end{array}$$ (17)

Equation 17 applies the IFFT to the masked spectrum *E*_*i*_[*k*]; *A*_*i*_[*n*] is the *i*^*th*^ FIBF, indicating the time-domain oscillatory component of the MI-EEG restricted to subband *i*.

The conventional FFT-based filter bank and the proposed FDM-based approach differ primarily in how the decomposed components are defined and represented. A conventional FFT filter bank applies predefined fixed frequency bands to obtain band-limited signals. In contrast, the original FDM (Singh et al., 2017) is a data-driven decomposition framework that determines frequency boundaries according to the spectral characteristics of the signal and represents the resulting components as FIBFs. In this work, the FDM principle is specifically adapted to MI-EEG by restricting the decomposition to the MI-relevant 8–30 Hz range and using eight subbands. For each subband, the corresponding FFT components are selected and reconstructed using the IFFT to obtain individual time-domain FIBFs. These channel-wise FIBFs are then provided to the 1D-CNN as separate spectral components for MI-EEG classification. The overall Fourier Decomposition process for MI-EEG is depicted in Figure 5. The input EEG signal was first band-pass filtered and then decomposed into its frequency domain using the FFT. The resulting spectrum is divided into subbands using binary masks, and each subband is subsequently reconstructed into an FIBF using the IFFT.

**Figure 5:**
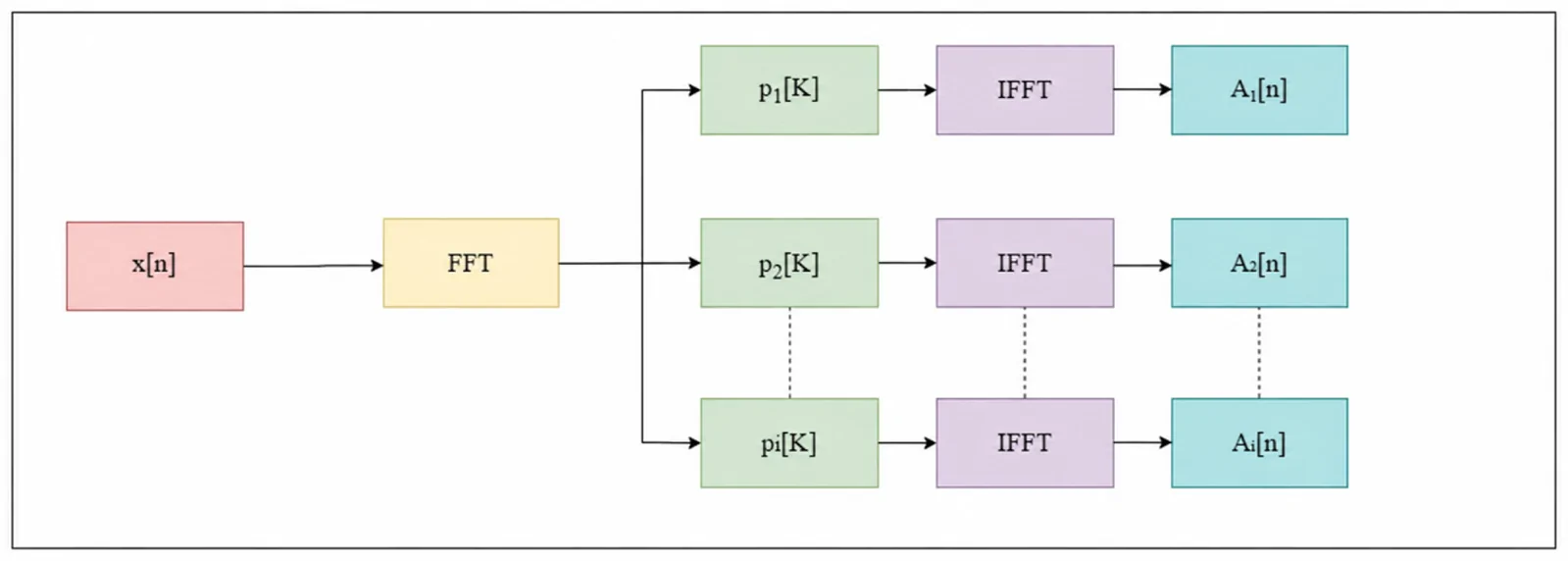
Workflow of the Fourier decomposition method (FDM) for MI-EEG processing using bandpass and FFT.

### 3.4 Proposed CNN model

The proposed SW-FDM-based 1D-CNN model as depicted in Figure 6 is designed to achieve reliable classification performance for MI-EEG tasks by effectively learning features from FDM-based representations. The architecture comprises two sequential convolution blocks, one Adaptive Average Pooling block, and a fully connected block used for classification purposes. Every convolutional block incorporates a 1D convolution layer, batch normalization, and a ReLU activation function, enabling feature extraction, normalization, and nonlinear representation learning. Following FDM decomposition, each EEG channel is decomposed into multiple FIBFs. The resulting FIBFs from all EEG channels are arranged to form the input to the 1D-CNN, where each channel FIBF combination is treated as an individual input feature map. The 1D convolution kernels are applied over the temporal dimension to learn temporal representations of the decomposed EEG signals. The information corresponding to each EEG channel is retained through these individual feature maps, while no explicit spatial convolution is performed across the EEG channels. Table 1 summarizes the layer details of the implemented 1D CNN used for MI-EEG classification. Here, *T*, *F*, *C*, *K* denote the window length, number of FIBFs, channels, and classes for binary as well as multi-class classification, respectively. The first convolutional block employs 64 filters with a kernel size of 7 to capture broad temporal dependencies and local temporal patterns from the FDM-based EEG representations. The relatively larger kernel is intended to capture broader temporal characteristics from adjacent EEG samples, while 64 filters provide adequate representational capacity for learning low-level discriminative features. Batch normalization follows each convolutional operation to stabilize feature distributions during training and facilitate faster convergence. Nonlinear feature learning is achieved through the ReLU activation function, allowing the model to capture complex representations.

**Figure 6:**
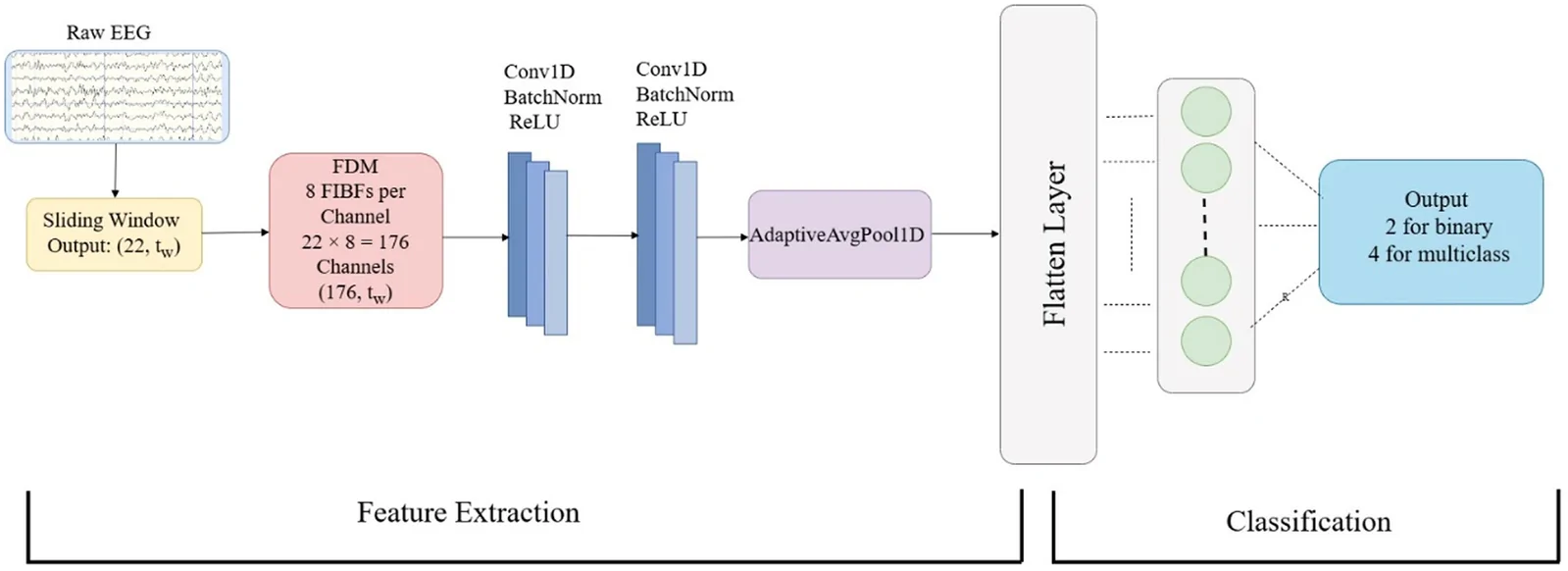
Overview of the proposed 1D CNN framework for MI-EEG classification with SW-FDM feature extraction.

**Table 1:** Architecture details of the proposed 1D-CNN model.

Layer	#Filters	Size
Input	–	(*F* · *C, t*_*w*_)
Conv1D layer 1	64	7
Batch normalization	–	–
ReLU activation	–	–
Conv1D layer 2	128	5
Batch normalization	–	–
ReLU activation	–	–
Adaptive average pooling	–	1
Flatten	–	–
Dense	–	*K*

The second convolutional block employs 128 filters with a kernel size of 5 to refine the features extracted by the preceding convolutional layer and learn higher-level temporal representations. The smaller kernel focuses on learning finer local temporal structures, whereas increasing the filter count from 64 to 128 allows the model to learn richer hierarchical feature representations. As in the first convolutional block, batch normalization improves training stability and accelerates convergence, followed by ReLU activation to model nonlinear relationships and enhance hierarchical feature learning. After feature extraction, the model employs Adaptive Average Pooling to lower model complexity and mitigate overfitting. The output generated by the Adaptive Average Pooling is reshaped into a one-dimensional feature vector using a flatten operation. The classification block comprises a fully connected layer that maps the extracted features to *K* motor imagery classes (two for binary and four for multi-class classification). Model optimization employed the Adam optimizer and the CrossEntropy loss function. Training was carried out for 100 epochs using a batch size of 64.

### 3.5 Experimental protocol

The classification performance for binary and multiclass classification was evaluated using two experimental paradigms: subject-dependent and subject-independent. For subject-dependent evaluation, a trial-level segregation was performed by partitioning the unique trial indices prior to any temporal processing, such that samples associated with the same trial were not included in both the training and testing sets. For subject-independent evaluation, one subject was retained for testing, whereas the rest of the subjects were used for training. To prevent cross-subject information leakage, subject-wise separation was maintained between the source subjects and the held-out target subject, while the target subject's trials were partitioned into calibration and testing sets. After splitting, SW segmentation was performed exclusively within the boundaries of each isolated trial. Because the sliding window boundaries were strictly confined within their respective trials and never bridged across them, overlapping windows from a single trial were completely restricted to a single partition. This strategy ensures strict separation between training and testing samples, thereby avoiding data leakage.

## 4 Results and discussion

The BCIC IV 2a dataset includes EEG data acquired from nine subjects (S1–S9) who performed MI activities. It comprises four MI tasks: tongue, left hand, right hand, and foot movements. The BCIC III IVa dataset includes EEG data from five subjects aa, al, av, aw, and ay, who were directed to perform MI activities, and it comprises two MI tasks, namely, right-hand and foot movements. Feature extraction is vital for enhancing performance. PyTorch was used for implementation, training, and evaluation. The proposed pipeline integrates SW-based segmentation, FDM-based feature extraction, and 1D-CNN. The predictions from all windows in a single trial are aggregated via majority voting to determine a final label.

### 4.1 Multiclass and binary subject dependent evaluation

For multiclass subject-dependent classification, as represented in Table 2, an average accuracy of 79.14 ± 12.82%, a precision of 80.33 ± 10.92%, and a recall of 79.09 ± 12.25% indicate strong classification performance across the four MI classes. The F1-score of 0.78 ± 0.12 indicates the model's consistency across subjects and classes. The specificity of 92.98 ± 4.19% highlights the ability of the classifier to reduce FPs. Subjects 1 and 8 achieved high classification performance, whereas Subject 6 exhibited comparatively lower performance.

**Table 2:** Subject-dependent multiclass classification performance on the BCIC IV 2a dataset.

Subject	Accuracy (%)	Precision (%)	Recall (%)	Specificity (%)	F1-score
S1	90.91	91.22	90.80	96.97	0.90
S2	67.01	66.38	67.03	88.90	0.66
S3	85.18	85.12	85.03	95.08	0.85
S4	64.15	71.69	64.49	88.18	0.63
S5	81.13	81.94	81.18	93.70	0.80
S6	59.09	63.33	60.15	86.29	0.60
S7	87.27	87.00	86.95	95.76	0.86
S8	96.22	94.64	94.51	98.17	0.94
S9	81.25	81.70	81.54	93.75	0.81
**Average**	**79.14** **±12.82**	**80.33** **±10.92**	**79.09** **±12.25**	**92.98** **±4.19**	**0.78** **±0.12**

Bold values indicate the results obtained by the proposed method and Percentage Accuracy of proposed work.

For multiclass classification, illustrated by the confusion matrix in Figure 7, 92.86% of the left-hand, 100% of the right-hand, 85.71% of both-feet, and 84.62% of the tongue trials were successfully classified. Among the four classes, the right-hand class exhibited the highest accuracy. A small number of misclassifications were observed for all classes. Out of 100% of left-hand trials, 92.86% were accurately classified as left hand, while 7.14% were misclassified as right hand.

**Figure 7:**
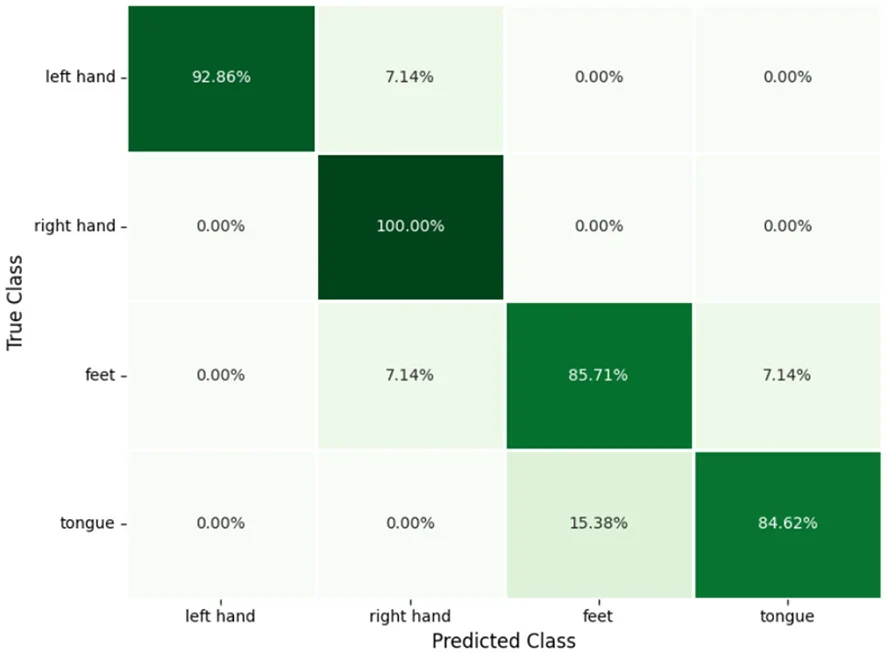
Confusion matrix on the BCIC IV 2a dataset.

For binary subject-dependent classification, as represented in Table 3, an average accuracy of 93.67 ± 9.91%, a precision of 96.67 ± 7.46%, and a recall of 89.37 ± 15.69% indicate strong classification performance across the two MI classes. An F1-score of 0.92 ± 0.12 indicates the model's consistency across subjects and classes. The specificity of 97.78% highlights the classifier's ability to reduce FPs. Subjects aw and ay achieved the highest classification accuracies of 100% and 100%, respectively, whereas subject av exhibited comparatively lower performance.

**Table 3:** Subject-dependent binary classification performance on the BCIC III IVa dataset.

Subject	Accuracy (%)	Precision (%)	Recall (%)	Specificity (%)	F1-score
aa	94.12	100	88.89	100	0.94
al	97.77	100	95.45	100	0.97
aw	100	100	100	100	1.0
av	76.47	83.33	62.50	88.89	0.71
ay	100	100	100	100	1.0
**Average**	**93.67** **±9.91**	**96.67** **±7.46**	**89.37** **±15.69**	**97.78** **±4.97**	**0.92** **±0.12**

Bold values indicate the results obtained by the proposed method and Percentage Accuracy of proposed work.

For binary classification, the two classes, as illustrated by the confusion matrix in Figure 8, show that 98.28% of the right hand and 89.29% of the foot trials were successfully classified with consistent classification. Among the two, the right-hand class exhibited the highest level of accuracy. The misclassification rates were minimal for all classes, although all were below 11%. Out of 100% of right-hand trials, 98.28% were accurately classified as right hand, and 1.72% were misclassified as foot.

**Figure 8:**
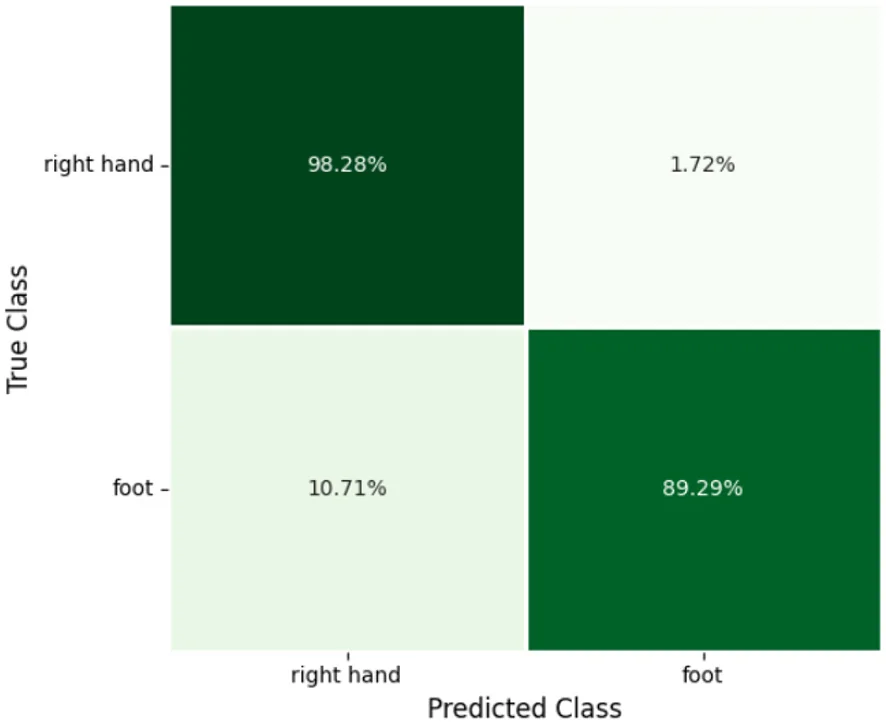
Confusion matrix on the BCIC III IVa dataset.

### 4.2 Binary and multiclass subject-independent evaluation

The proposed framework was evaluated under a subject-independent setting using a calibration-based LOSO protocol. For each fold, the model was pretrained using the source subjects and subsequently fine-tuned using 20% of the trials from the unseen target subject as calibration data. The remaining 80% of the target subject's trials were used exclusively for testing. Thus, the adopted LOSO protocol represents a calibration-based subject-independent evaluation rather than a strict zero-shot setting, since target-subject data were used for model adaptation. To ensure a fair evaluation, the target subject's data were partitioned at the trial level before sliding-window segmentation, preventing overlapping windows from the same trial from appearing in both the calibration and testing sets. To provide a more reliable assessment of cross-subject generalization, the LOSO results are reported not only in terms of average classification accuracy but also with the corresponding standard deviation across the held-out subjects. This variability measure reflects the extent of inter-subject performance differences, which are particularly important in MI-EEG classification because of substantial physiological and signal-distribution variability among subjects. For the BCIC III IVa dataset, the proposed framework achieved an average LOSO classification accuracy of 76.49 ± 14.74%, whereas for the BCIC IV-2a dataset, an average accuracy of 55.50 ± 19.01% was obtained. The larger standard deviation observed for BCIC IV-2a indicates greater inter-subject variability in the four-class MI task. The corresponding subject-wise results are presented in Tables 4, 5, while Table 6 summarizes the mean ± standard deviation of the proposed framework and the reimplemented baseline models under the same LOSO protocol. In addition, Table 7 presents the subject-wise multiclass LOSO accuracies together with the paired t-test and Wilcoxon signed-rank test results, thereby complementing the variability analysis with statistical significance testing.

**Table 4:** Subject-wise LOSO classification performance on the BCIC III IVa dataset.

Test subject	Accuracy (%)	Precision (%)	Recall (%)	Specificity (%)	F1-score
aa	78.57	79.55	79.55	77.50	0.79
al	91.96	97.00	86.61	97.32	0.91
av	61.90	60.42	69.05	54.76	0.64
aw	89.29	88.46	88.46	90.00	0.88
ay	60.71	47.06	80.00	50.00	0.59
**Average**	**76.49** **±14.74**	**81.36** **±20.48**	**81.65** **±7.63**	**81.56** **±20.97**	**0.81** **±0.14**

Bold values indicate the results obtained by the proposed method and Percentage Accuracy of proposed work.

**Table 5:** Subject-wise LOSO classification performance on the BCIC IV 2a dataset.

Test subject	Accuracy (%)	Precision (%)	Recall (%)	Specificity (%)	F1-score
S1	72.40	72.35	72.38	90.80	0.72
S2	36.51	36.54	36.41	78.84	0.36
S3	83.60	83.80	83.48	94.53	0.83
S4	42.39	44.25	42.47	80.78	0.43
S5	33.70	36.63	34.02	78.04	0.34
S6	40.26	39.38	39.55	79.87	0.39
S7	52.11	55.63	51.42	84.02	0.53
S8	78.92	79.20	78.74	92.97	0.79
S9	59.64	59.77	59.95	86.51	0.60
**Average**	**55.50** **±19.01**	**56.00** **±18.57**	**55.50** **±19.02**	**85.15** **±6.34**	**0.55** **±0.19**

Bold values indicate the results obtained by the proposed method and Percentage Accuracy of proposed work.

**Table 6:** Average LOSO classification accuracies (%) of the reimplemented models under a unified experimental framework.

Model	BCIC III IVa (%)	BCIC IV-2a (%)
EEGNet (Lawhern et al., 2018)	71.48 ± 12.20	54.05 ± 15.28
FBCNet (Mane et al., 2021)	69.06 ± 9.94	48.02 ± 12.83
TCANet (Zhao et al., 2025)	57.86 ± 6.01	33.17 ± 7.43
EEG Conformer (Song et al., 2023)	75.88 ± 11.16	45.10 ± 14.94
Min2Net (Autthasan et al., 2022)	68.40 ± 7.27	38.24 ± 10.05
SST-DPN (Han et al., 2025)	69.71 ± 13.89	54.73 ± 18.40
SCLDGN (Zhi et al., 2024)	70.87 ± 21.74	49.55 ± 16.97
EEG-DG (Song et al., 2025)	70.60 ± 10.60	38.34 ± 14.38
**Proposed**	**76.49 ± 14.74**	**55.50 ± 19.01**

Bold values indicate the results obtained by the proposed method and Percentage Accuracy of proposed work.

**Table 7:** Subject-wise multiclass LOSO classification accuracies (%) and statistical comparison of the proposed framework with competing methods on the BCIC IV-2a dataset.

Test subject	EEGNet (Lawhern et al., 2018)	FBCNet (Mane et al., 2021)	Min2Net (Autthasan et al., 2022)	TCANet (Zhao et al., 2025)	EEG conformer (Song et al., 2023)	SST-DPN (Han et al., 2025)	SCLDGN (Zhi et al., 2024)	EEG-DG (Song et al., 2025)	Proposed
S1	69.27	53.56	51.60	40.64	64.06	69.86	62.10	39.73	72.40
S2	43.39	33.80	27.78	27.31	25.93	33.33	31.48	26.39	36.51
S3	79.37	68.32	53.70	34.72	54.50	81.94	78.70	50.46	83.60
S4	45.11	42.03	40.48	25.24	41.30	37.61	36.66	35.71	42.39
S5	38.04	32.83	23.33	25.71	30.98	34.28	33.33	23.81	33.70
S6	43.51	39.93	35.23	32.39	32.47	44.31	38.06	38.07	40.26
S7	55.26	41.37	32.72	27.19	35.79	52.33	41.01	17.05	52.11
S8	70.81	61.12	41.98	40.09	64.86	72.64	64.62	58.02	78.92
S9	41.69	59.19	37.37	45.26	56.02	66.31	60.00	55.79	59.64
**Mean** **±SD**	54.05 ± 15.28	48.02 ± 12.83	38.24 ± 10.05	33.17 ± 7.43	45.10 ± 14.94	54.73 ± 18.40	49.55 ± 16.97	38.34 ± 14.38	**55.50 ± 19.01**
**Wilcoxon** **p**	0.9102	**0.0039**	**0.0039**	**0.0039**	**0.0039**	0.6523	**0.0078**	**0.0039**	–
**Paired** **t-test** **p**	0.5930	**0.0242**	**0.0022**	**0.0019**	**0.0070**	0.5932	**0.0075**	**0.0050**	–

Statistical comparisons were performed between the proposed framework and each competing method using the subject-wise LOSO accuracies of nine subjects (*n* = 9). Bold *p*-values indicate statistically significant differences at *p* < 0.05.

For the binary motor imagery classification on the BCIC III IVa dataset, the proposed framework attained an average accuracy of 76.49 ± 14.74 %, with corresponding precision, recall, specificity, and F1-score of 81.36± 20.48 %, 81.65± 7.63 %, 81.56± 20.97 %, and 0.81 ± 0.14, respectively, as summarized in Table 4. Subject-wise performance varied across the five subjects, with al achieving the highest accuracy of 91.96%, whereas ay obtained the lowest accuracy of 60.71%, highlighting the influence of inter-subject variability. Furthermore, al recorded the highest precision of 97.00% and specificity of 97.32%, while ay achieved the highest recall of 80.00%. These results demonstrate that the proposed framework effectively adapts to unseen subjects while maintaining a rigorous leak-free evaluation.

The same evaluation protocol was applied to the four-class MI-EEG classification on the BCIC IV-2a dataset, which is inherently more challenging because of the four class setting and substantial inter-subject variability. As summarized in Table 5, the proposed framework attained an average accuracy of 55.50%, with corresponding precision, recall, F1-score, and specificity of 56.00%, 55.50%, 0.55%, and 85.15%, respectively. Subject-wise accuracies ranged from 33.70% for S5 to 83.60% for S3, with S3, S8, and S1 exhibiting the best performance, while S5, S2, S6, and S4 showed comparatively lower accuracies owing to larger inter-subject variability. Despite the challenges of four-class classification, the proposed framework maintained cross-subject performance under the same leak-free, calibration-based LOSO protocol.

### 4.3 Experimental validation

To further validate the proposed framework under the subject-independent setting, eight representative DL models, namely EEGNet (Lawhern et al., 2018), FBCNet (Mane et al., 2021), TCANet (Zhao et al., 2025), EEG Conformer (Song et al., 2023), Min2Net (Autthasan et al., 2022), SST-DPN (Han et al., 2025), SCLDGN (Zhi et al., 2024) and EEG-DG (Song et al., 2025) were reimplemented and evaluated exclusively using the LOSO protocol. For a fair comparison, all reimplemented baseline models were evaluated using identical preprocessing, augmentation, and training settings as the proposed framework. Specifically, all models used the same 8–30 Hz band-pass filtering, seven-window sliding-window-based data augmentation, FDM with eight FIBFs, LOSO evaluation protocol, Adam optimizer, CrossEntropy loss function, batch size of 64, source training for 100 epochs, and subject-specific calibration for 40 epochs. The left-out subject was partitioned at the trial level into 20% calibration data and 80% testing data before sliding-window segmentation to prevent data leakage. The average classification performance of the reimplemented models is reported in Table 6 as mean ± standard deviation across the held-out subjects. On the BCIC III IVa dataset, EEGNet (Lawhern et al., 2018), FBCNet (Mane et al., 2021), TCANet (Zhao et al., 2025), EEG Conformer (Song et al., 2023), Min2Net (Autthasan et al., 2022), SST-DPN (Han et al., 2025), SCLDGN (Zhi et al., 2024), and EEG-DG (Song et al., 2025) achieved accuracies of 71.48 ± 12.20%, 69.06 ± 9.94%, 57.86 ± 6.01%, 75.88 ± 11.16%, 68.40 ± 7.27%, 69.71 ± 13.89%, 70.87 ± 21.74%, and 70.60 ± 10.60%, respectively, whereas the proposed framework achieved the highest accuracy of 76.49 ± 14.74%.

On the more challenging BCIC IV 2a dataset, EEGNet (Lawhern et al., 2018), FBCNet (Mane et al., 2021), TCANet (Zhao et al., 2025), EEG Conformer (Song et al., 2023), Min2Net (Autthasan et al., 2022), SST-DPN (Han et al., 2025), SCLDGN (Zhi et al., 2024), and EEG-DG (Song et al., 2025) attained accuracies of 54.05 ± 15.28%, 48.02 ± 12.83%, 33.17 ± 7.43%, 45.10 ± 14.94%, 38.24 ± 10.05%, 54.73 ± 18.40%, 49.55 ± 16.97%, and 38.34 ± 14.38%, respectively, while the proposed framework achieved the highest mean accuracy of **55.50 ± 19.01%**. These results indicate that the proposed framework achieved the highest mean classification accuracy on both datasets, while the reported standard deviations reflect the inter-subject variability observed under the LOSO evaluation protocol.

First, we compare the proposed method with EEGNet (Lawhern et al., 2018), a compact convolutional neural network specifically designed for EEG-based BCI applications. EEGNet consists of temporal convolutional layers for spectral feature extraction, followed by depthwise convolutions that model spatial information across EEG channels. The resulting features are subsequently fused using separable convolutions to achieve computationally efficient feature learning. Owing to its lightweight architecture and competitive performance, EEGNet is widely adopted as a baseline model for EEG classification tasks. The proposed method is further compared with FBCNet (Mane et al., 2021), a CNN-based model for MI-EEG classification that employs filter-bank decomposition, depthwise spatial convolutions, and variance-based temporal feature extraction to learn discriminative spectro-spatial-temporal representations. TCANet Zhao et al., (2025) is also included for comparison. It integrates a multi-scale convolutional module, a temporal convolutional module, and a multi-head self-attention mechanism to progressively learn local, fused, and global spatiotemporal representations for MI-EEG decoding. In addition, the proposed method is compared with EEG Conformer (Song et al., 2023), a hybrid CNN-Transformer architecture that combines temporal and spatial convolutions with transformer-based multi-head self-attention to jointly learn local and global spatiotemporal representations for EEG classification. The proposed method is compared with MIN2Net (Autthasan et al., 2022), a multi-task learning framework for subject-independent MI-EEG classification. MIN2Net integrates a convolutional autoencoder, deep metric learning, and a supervised classifier to jointly learn compact and discriminative latent representations while simultaneously performing signal reconstruction and classification. Further we compare the proposed method with SST-DPN (Han et al., 2025), a lightweight framework designed to jointly model spatial–spectral and long-term temporal information. SST-DPN employs adaptive spatial–spectral fusion to capture relationships among EEG channels and spectral components, multi-scale variance pooling to model long-term temporal information, and dual prototype learning to enhance intra-class compactness and inter-class separation. Owing to its joint spatial–spectral–temporal representation learning, SST-DPN provides a representative recent baseline for comparison with the proposed framework. SCLDGN (Zhi et al., 2024) is further included as a recent domain-generalization baseline for cross-subject MI decoding. Its feature encoder applies multi-scale depthwise temporal convolutions followed by spatial convolution to extract discriminative spatio-temporal EEG representations. Deep CORAL-based domain alignment is employed to reduce distribution discrepancies among source subjects, while supervised contrastive learning with domain-agnostic mixup promotes class-level alignment and domain-invariant representation learning. Finally, the latent-distribution domain-generalization framework proposed in (Song et al. 2025) is considered. The method employs multiple EEGNet- and ShallowConvNet-based classification branches to learn diverse latent source-domain distributions. A score network estimates the relative contribution of individual branches, while a gating mechanism adaptively combines their outputs for final classification. This model therefore provides a representative recent multi-branch domain-generalization baseline for cross-subject MI-EEG decoding. The results indicate that the proposed approach attained better average classification accuracy compared against the reimplemented baseline models.

To further evaluate whether the observed differences in multiclass LOSO classification performance were statistically significant, a subject-wise statistical analysis was conducted on the BCIC IV-2a dataset. The classification accuracies obtained for the same nine held-out subjects were compared between the proposed framework and EEGNet (Lawhern et al., 2018), FBCNet (Mane et al., 2021), Min2Net (Autthasan et al., 2022), TCANet (Zhao et al., 2025), EEG Conformer (Song et al., 2023), SST-DPN (Han et al., 2025), SCLDGN (Zhi et al., 2024), and EEG-DG (Song et al., 2025). Both the paired *t*-test and the Wilcoxon signed-rank test were employed. The paired *t*-test evaluates the mean difference between paired subject-wise accuracies under the parametric assumption of normally distributed paired differences, whereas the Wilcoxon signed-rank test provides a complementary non-parametric assessment that does not require normality of the paired differences. Thus, the use of both tests provides complementary statistical evidence regarding the observed performance differences. A significance level of *p* < 0.05 was considered statistically significant. For BCIC IV 2a, as summarized in Table 7, both tests yielded consistent conclusions. Significant differences were observed for FBCNet (Mane et al., 2021), Min2Net (Autthasan et al., 2022), TCANet (Zhao et al., 2025), EEG Conformer (Song et al., 2023), SCLDGN (Zhi et al., 2024), and EEG-DG (Song et al., 2025), whereas the comparisons with EEGNet (Lawhern et al., 2018) and SST-DPN (Han et al., 2025) were not significant. These results provide statistical support for the multiclass LOSO improvements over the majority of the evaluated methods. For BCIC III IVa, the paired *t*-test indicated a significant improvement over EEGNet (Lawhern et al., 2018) (*p* = 0.0467), while the remaining comparisons were not significant (*p* > 0.05). The Wilcoxon signed-rank test did not reveal significant differences for any comparison. Nevertheless, the proposed framework achieved the highest mean accuracy of 76.49% across the five subjects.

### 4.4 Ablation study

#### 4.4.1 Effect of FIBFs

To identify the most favorable configuration for MI-EEG classification, an ablation study was conducted by incorporating different number of FIBFs. To systematically determine the appropriate number of FIBFs, experiments were conducted using 6, 8, 10, and 12 FIBFs while keeping the remaining experimental settings unchanged. The BCIC IV 2a dataset, which is a multiclass classification dataset, was evaluated for the FDM components under the subject-dependent evaluation protocol. The EEG signals were decomposed into selected FIBFs using FDM. Features were extracted from the decomposed signals using a 1D-CNN. Table 8 shows a comparative evaluation for various number of FIBFs for the BCI IV 2a and III IVa dataset. Based on the analysis performed, the configuration with eight FIBFs yielded the highest multiclass classification accuracy of 79.14 ± 12.83 %. The selection of eight FIBFs is supported by both empirical evaluation and the physiological characteristics of motor imagery EEG. MI primarily modulates the μ (8–12 Hz) and β (13–30 Hz) rhythms through event-related desynchronization (ERD) as well as event-related synchronization (ERS). Therefore, eight FIBFs provide sufficient spectral resolution to effectively capture these physiologically relevant frequency components. Using fewer FIBFs may result in inadequate spectral separation, whereas increasing the number beyond eight may introduce unnecessary spectral fragmentation without improving classification performance. When using six FIBFs, the multiclass classification accuracy was comparatively lower, resulting in 72.70 ± 14.16%. This indicates inadequate spectral decomposition, leading to reduced feature quality. For 10 FIBFs, the accuracy was 72.38 ± 11.80%, which is slightly less than that of 6 FIBFs, resulting in a further decrease in accuracy. Using 12 FIBFs for multiclass classification resulted in 73.98± 11.88%, which slightly improved over 6 and 10 FIBFs but did not outperform 8 FIBFs. Eight FIBFs provided a better balance compared to under-decomposition with 6, 10, and 12 FIBFs, ensuring adequate spectral separation while excluding noise.

**Table 8:** Impact of the FIBF count on MI-EEG classification accuracy for the BCIC IV-2a and BCI III IVa datasets.

No. of FIBF	BCIC IV 2a accuracy (%)	BCIC III IVa accuracy (%)
6	72.70 ± 14.16	91.91 ± 12.45
**8**	**79.14** **±12.38**	**93.67** **±9.91**
10	72.38 ± 11.80	90.14 ± 15.03
12	73.98 ± 11.88	92.49 ± 10.19

Bold values indicate the results obtained by the proposed method and Percentage Accuracy of proposed work.

From the experimental analysis, the configuration with eight FIBFs yielded the highest binary classification accuracy of 93.67 ± 9.91%. When using six FIBFs, the classification accuracy was comparatively lower, resulting in 91.91± 12.45 %. This indicates inadequate spectral decomposition, leading to reduced feature quality. For 10 FIBFs, the accuracy was 90.14 ± 15.03 %, which is slightly less than that of 6 FIBFs, resulting in a further decrease in accuracy. Using 12 FIBFs, binary classification accuracy was 92.49 ± 10.19 % and slightly improved over 6 and 10 FIBFs but did not outperform 8 FIBFs. Eight FIBFs provided a better balance compared to 6, 10 and 12 FIBFs, ensuring adequate spectral separation with the exclusion of noise. As shown in Table 7, eight FIBFs achieved the highest average accuracy on both datasets, with 79.14 ± 12.83% on BCI Competition IV-2a and 93.67 ± 9.91% on BCI Competition III IVa. The results indicate that eight FIBFs provide a suitable balance between spectral resolution and decomposition complexity. Therefore, eight FIBFs were selected for the subsequent experiments.

#### 4.4.2 Comparison with conventional FFT-based filter bank

To further evaluate the effectiveness of the proposed FDM-based decomposition, a comparison was performed with a conventional FFT-based filter bank under identical experimental conditions as presented in Table 9. Both methods used the same 8–30 Hz frequency range, eight frequency bands, seven-window sliding-window configuration, and 1D-CNN architecture. The conventional FFT-based filter bank achieved average accuracies of 89.75 ± 12.56% and 70.35 ± 15.77% on BCIC III IVa and BCIC IV-2a, respectively, whereas the proposed FDM-based approach achieved 93.67 ± 9.91% and 79.14 ± 12.82%. Thus, the proposed approach improved the average accuracy by 3.92% and 8.79%.

**Table 9:** Comparison of FDM-based decomposition and FFT-based filter bank performance.

Dataset	FDM-based decomposition accuracy (%)	FFT-based filter bank (%) accuracy
BCIC III IVa	**93.67 ± 9.91**	89.75 ± 12.56
BCIC IV 2a	**79.14 ± 12.82**	70.35 ± 15.77

#### 4.4.3 Architectural contribution analysis

A progressive ablation study was conducted for the BCIC IV 2a and BCIC III IVa dataset under the subject-dependent evaluation protocol, as shown in Table 10. For the BCIC IV 2a, when raw EEG signals were directly provided to the 1D-CNN, the classification accuracy was confined to 52.29 ± 14.48%, confirming that direct trial-level processing limits effective feature discrimination. Introducing sliding window (SW) segmentation considerably enhanced the performance to 70.19 ± 14.41%, highlighting the importance of localized temporal representation in enhancing the discriminative feature learning. The improvement with SW is attributed to the division of each EEG trial into multiple overlapping segments, which provides localized temporal information and additional training samples for the 1D-CNN. To independently assess the contribution of FDM, raw EEG with FDM and 1D-CNN without SW was evaluated and achieved an accuracy of 67.49 ± 16.83%. The improvement with FDM is attributed to the decomposition of EEG signals into frequency-specific FIBFs, which enhances spectral representation and provides more discriminative frequency information to the 1D-CNN. The highest accuracy of 79.14 ± 12.83% was achieved when FDM-based decomposition was incorporated with SW and 1D-CNN. The results indicate that FDM strengthens spectral separability, leading to more informative feature extraction. Overall, the steady performance increase (26.85% improvement from raw input to the complete pipeline) validates the integrated combination of SW segmentation and FDM decomposition with a 1D-CNN.

**Table 10:** Component-wise ablation study of the proposed SW–FDM–1D CNN framework.

Dataset	FDM-based decomposition accuracy (%)	FFT-based filter bank (%) accuracy
Raw EEG + 1D-CNN	52.29 ± 14.48	81.71 ± 14.14
Raw EEG + FDM + 1D-CNN	67.49 ± 16.83	86.22 ± 19.73
Raw EEG + SW + 1D-CNN	70.19 ± 14.41	90.14 ± 15.03
Raw EEG + SW + FDM + 1D-CNN	**79.14** **±12.83**	**93.67** **±9.91**

Bold values indicate the results obtained by the proposed method and Percentage Accuracy of proposed work.

A similar observation was made for the BCIC III IVa dataset, where the accuracy improved from 81.71 ± 14.14% with raw EEG and 1D-CNN to 90.14 ± 15.03% with SW and raw EEG with FDM and 1D-CNN without SW achieved 86.22 ± 19.73%, demonstrating the independent contribution of FDM-based spectral decomposition, and reached 93.67 ± 9.91% when FDM was incorporated, resulting in an 11.96% improvement from the baseline to the complete pipeline. These results indicate that the integration of SW and FDM with 1D-CNN enhances MI-EEG classification.

### 4.5 Comparison with SOTA methods

Table 11 provides a comparison of diverse SOTA techniques, such as decomposition-based, spatial filtering–based, statistical, Riemannian, and DL approaches for MI-EEG in subject-dependent binary classification, which demonstrate improved results compared with numerous SOTA techniques. It achieved 100% accuracy for the *aw* and *ay* subjects.

**Table 11:** Comparison with SOTA methods for BCIC III IVa binary MI-EEG classification.

Ref	Methodology	aa	al	av	aw	ay	Avg. Acc. (%)
Kevric and Subasi (2017)	WPD	96	92.3	88.9	95.4	91.4	92.8
Belwafi et al. (2018)	Adaptive FB + LDA	66.8	96.1	52.1	71.4	50	67.3
Dokur and Olmez (2021)	CNN	85.4	60	65.4	83.6	72.7	73.4
Sadiq et al. (2019)	R-CSP	76.8	98.2	74.5	92.9	77	83.9
Park and Chung (2018)	CSP	100	74.1	67.8	90	89.2	84.4
Singh et al. (2019)	Riemannian	81.25	100	76.53	87.05	91.26	87.21
Park and Chung (2020)	FBCSP	98.21	89.29	73.49	92.86	89.29	88.6
Jin et al. (2020)	CSP	82.1	95.0	72.1	90.7	91.8	86.3
Abbasi et al. (2026)	CNN-transformer	90.08	95.96	92.19	91.00	93.99	92.64
**Proposed**		**94.12**	**97.77**	**76.47**	**100**	**100**	**93.67**

Bold values indicate the results obtained by the proposed method and Percentage Accuracy of proposed work.

Although Kevric and Subasi (2017) achieved competitive performance of 92.8%, it relied on handcrafted statistical features using kNN classification, which limited the learning capacity to automatically learn deep representations. The adaptive filter bank with LDA (Belwafi et al., 2018) achieved an accuracy of 67.3% because the model was restricted in capturing nonlinear and complex patterns from the signal. The CNN-based approach (Dokur and Olmez, 2021) achieved an accuracy of 73.4%; however, the framework was trained without explicit signal transformation and augmentation. The R-CSP (Sadiq et al., 2019) and CSP (Park and Chung, 2018) methods attained accuracies of 83.9% and 84.4%, respectively, but they relied on handcrafted features. The Riemannian approach (Singh et al., 2019) achieved a mean accuracy of 87.21%, although it involved computationally expensive manifold operations. The FBCSP (Park and Chung, 2020) achieved an accuracy of 88.6%; however, the approach is computationally overhead as it calculates the fisher score across multiple channels. Although Jin et al. (2020) achieved an accuracy of 86.3% using bi-spectrum-based channel selection, the improvement was not consistent across all subjects. The CNN-Transformer (Abbasi et al., 2026) achieved an accuracy of 92.64%; however, the architecture relies on transformer-based attention that increases the model complexity. In contrast, the proposed methodology obtained a higher mean accuracy of 93.67%, demonstrating the performance of the proposed framework.

Table 12 presents a comparison of diverse SOTA techniques for subject-dependent multiclass MI-EEG classification. Most current methods use time-series EEG signals as inputs, employing DL approaches such as CNN + FBCSP (Sakhavi et al., 2018), CNN + LSTM (Lu et al., 2019), CNN + CSP (Yang et al., 2015), GRU (Qiao and Bi, 2019), EEGNet (Riyad et al., 2021), ConvNet (Zhao et al., 2019). Although these methods achieve moderate classification accuracies between 68.45% and 78.81%, their performances remain limited. ShallowCNN (Liao et al., 2020), which utilizes EEG data as 2D images, achieved an accuracy of 74.6%. NFBCSP (Xu et al., 2022), CSP (Hersche et al., 2018), and Riemannian covariance-based methods (Yang et al., 2020) rely on manually designed features, which may limit feature learning from EEG signals. In contrast, the proposed methodology, which also operates on time-series EEG data, achieved an accuracy of 79.14%, indicating an improvement over existing approaches and validating the performance of the proposed feature extraction and classification framework for multiclass MI-EEG decoding.

**Table 12:** Comparison with SOTA methods for multiclass MI-EEG classification.

References	Methodology	Input representation	Accuracy (%)
Sakhavi et al. (2018)	CNN + FBCSP	Time-series EEG signal	74
Lu et al. (2019)	CNN + LSTM	Time-series EEG signal	76.6
Yang et al. (2015)	CNN + CSP	Time-series EEG signal	68.45
Qiao and Bi (2019)	GRU	Time-series EEG signal	74
Riyad et al. (2021)	EEGNet	Time-series EEG signal	74
Zhao et al. (2019)	ConvNet	Time-series EEG signal	69
Liao et al. (2020)	ShallowCNN	2D image	74.6
Xu et al. (2022)	NFBCSP	Time-series EEG signal	76.8
Hersche et al. (2018)	CSP	Time-series EEG signal	73.70
Yang et al. (2020)	Riemannian covariance	Time-series EEG signal	76.00
**Proposed**			**79.14**

Bold values indicate the results obtained by the proposed method and Percentage Accuracy of proposed work.

### 4.6 Comparative performance and model complexity analysis

Table 13 presents a comparison of the developed model with existing MI-EEG classification methods in terms of classification accuracy and parameter count. Computational complexity was assessed using floating-point operations (FLOPs), multiply-accumulate operations (MACs), and the number of learnable parameters for binary as well as multi-class classification tasks. For binary and multiclass classification, the model consists of approximately 0.12 million learnable parameters. For binary classification, 23.53 million MACs are required, which corresponds to 46.50 million FLOPs, whereas for multiclass classification, 83.83 million MACs are required, which corresponds to 167.05 million FLOPs. It was observed that there was an increase in the computational cost for multiclass compared with binary classification.

**Table 13:** Comparison of models emphasizing parameter efficiency for BCIC IV 2a.

Model	Accuracy (%)	Parameters (K)	MACs
RGN (Shi et al., 2024)	80.5	1,683K	–
EEGNet (Lawhern et al., 2018)	78.59	2,416K	–
CSP (Hersche et al., 2018)	73.70	261K	104M
DFFN (Li et al., 2019)	79.71	1,070K	132M
MSFBCNN (Wu et al., 2019)	75.80	155K	202M
CP-MixedNet (Chen et al., 2024)	67.17	836K	-
TS-SEFFNET (Oikonomou et al., 2020)	74.71	282K	–
MTFB-CNN (Li et al., 2023)	76.07	96,373K	–
ConvNet (Schirrmeister et al., 2017)	65.89	40,644K	–
Tensor-CSPNet (Ju and Guan, 2022)	74.92	27,104K	–
DJDAN (Hong et al., 2021)	76.52	150K	–
**Proposed**	**79.14**	**120K**	**83.83M**

Bold values indicate the results obtained by the proposed method and Percentage Accuracy of proposed work.

Table 13 presents a comprehensive comparison with SOTA models. The proposed model attained 79.14% accuracy with only 120K learnable parameters, exhibiting remarkable parameter efficiency. Compared with RGN (Shi et al., 2024) and DFFN (Li et al., 2019), which require 1683K and 1070K parameters, respectively, the proposed model achieves parameter reductions of 14.03 × and 8.92 × while maintaining competitive classification performance. Relative to other CNN-based models, parameter reductions of 2.18 ×, 1.29 ×, 7 ×, 2.35 ×, and 1.25 × were achieved compared with CSP (Hersche et al., 2018), MSFBCNN (Wu et al., 2019), CP-MixedNet (Chen et al., 2024), TS-SEFFNET (Oikonomou et al., 2020), and DJDAN (Hong et al., 2021), respectively, while achieving higher classification accuracy. Furthermore, substantial parameter reductions of 803.11 ×, 20.13 ×, 338.70 ×, and 225.87 × were obtained relative to MTFB-CNN (Li et al., 2023), EEGNet (Lawhern et al., 2018), ConvNet (Schirrmeister et al., 2017), and Tensor-CSPNet (Ju and Guan, 2022), respectively. Overall, the experimental results indicate that the proposed architecture maintains competitive classification accuracy with low computational overhead, making it appropriate for deployment in BCI systems operating under limited hardware resources.

### 4.7 Embedded implementation and performance evaluation

The computational performance of the proposed framework was tested out on the NVIDIA Jetson Nano. The platform, which includes a quad-core ARM Cortex-A57 CPU and a 128-core NVIDIA Maxwell GPU, enables efficient DL inference in resource-limited embedded systems. The model was implemented using PyTorch and tested through offline inference experiments according to the LOSO evaluation method. During each LOSO fold, the model corresponding to the left-out subject was loaded onto the Jetson Nano, and inference was performed using the complete offline processing pipeline. The pipeline consisted of band-pass filtering, SW segmentation, FDM based feature extraction, 1D-CNN inference, and final decision aggregation. Table 14 presents the trial-level computational latency and throughput of the proposed framework on the NVIDIA Jetson Nano.

**Table 14:** Trial-level computational latency and throughput of the proposed framework on the NVIDIA Jetson Nano.

Processing stage	BCIC III IVa	BCIC IV 2a
Band-pass filtering	7.720 ms/trial	8.828 ms/trial
Sliding-window generation	0.835 ms/trial	5.976 ms/trial
FDM	32.641 ms/trial	22.913 ms/trial
1D-CNN inference	16.084 ms/trial	46.702 ms/trial
Trial-level decision aggregation	0.839 ms/trial	0.636 ms/trial
**Total computational latency** **Throughput**	**58.119 ms/trial** **17.21 trials/s**	**85.055 ms/trial** **11.76 trials/s**

Bold values indicate the results obtained by the proposed method and Percentage Accuracy of proposed work.

To assess deployment performance, window-level and trial-level computational latencies were measured in addition to the latency of individual processing stages. The window-level latency represents the computational time required to process one EEG window, whereas the trial-level latency represents the complete computational processing time required to obtain the final decision for one trial. The deployment results are summarized in Tables 14, 15. For the BCIC III IVa dataset, the proposed framework achieved an average classification accuracy of 76.49 ± 14.74 %, with a window-level computational latency of 8.182 ms and a trial-level computational latency of 58.119 ms, corresponding to throughputs of 122.22 windows/s and 17.21 trials/s, respectively. Similarly, for the BCIC IV 2a dataset, the framework attained an average classification accuracy of 55.50 ± 19.01 %, with a window-level computational latency of 12.242 ms and a trial-level computational latency of 84.479 ms, corresponding to throughputs of 81.69 windows/s and 11.84 trials/s.

**Table 15:** Deployment performance of the proposed 1D-CNN framework on NVIDIA Jetson Nano.

Performance metric	BCI III IVa	BCI IV-2a
Classification accuracy (%)	76.49 ± 14.74	55.50 ± 19.01
1D-CNN latency (ms/window)	2.290	6.672
Complete window-level computational latency (ms/window)	8.182	12.242
Window-level throughput (windows/s)	122.22	81.69
Complete trial-level computational latency (ms/trial)	58.119	85.055
Trial-level throughput (trials/s)	17.21	11.76

Tables 14, 15 summarize the computational performance of the proposed framework on the NVIDIA Jetson Nano under the offline evaluation setting. Table 14 reports the execution time of the individual processing stages together with the overall trial-level latency and throughput, whereas Table 15 provides a consolidated comparison of the window-level and trial-level computational performance. Since the experiments were conducted using prerecorded EEG data, the reported values represent computational processing latency on the Jetson Nano and do not include EEG acquisition or signal-accumulation time. However, they should be interpreted as offline computational performance metrics rather than as evidence of complete real-time online MI-BCI operation. Therefore, they should not be interpreted as the complete online end-to-end decision latency of a live MI-BCI system. Nevertheless, the measured computational latency and throughput support the feasibility of deploying the proposed framework on a low-power embedded platform for offline experiment.

The proposed 1D-CNN shows lower parameter, MAC, and memory requirements than the lightweight models considered in Table 16. Compared with ATCNet (Altaheri et al., 2022), the proposed model reduces the parameter count by 93.75%, MACs by 79.55%, peak RAM usage by 84.49%, and inference latency by 34.22%. Against WST-CNN (Buragohain et al., 2024), the corresponding reductions are 94.57%, 82.89%, 86.70%, and 47.53%, respectively. Similarly, compared with DB-ATCNet (Zhou et al., 2024), the proposed model achieves reductions of 95.76% in parameters, 86.48% in MACs, 88.77% in peak RAM usage, and 55.52% in inference latency. The results for ATCNet (Altaheri et al., 2022), WST-CNN (Buragohain et al., 2024), and DB-ATCNet (Zhou et al., 2024) were taken from the reimplementation reported by MAML-LNN (Raza and Yusoff, 2026), where the models were reproduced using their official codebases and evaluated under a common experimental setting. Compared with MAML-LNN (Raza and Yusoff, 2026), the proposed 1D-CNN requires 85.37% fewer parameters, 50.69% fewer MACs, and 66.76% less peak RAM, its inference latency is 79.62% higher. Overall, the proposed 1D-CNN demonstrates substantially lower computational and memory requirements, supporting its suitability for deployment on the NVIDIA Jetson Nano.

**Table 16:** Computational efficiency comparison with representative lightweight models on BCIC IV 2a.

Model	Parameters (M)	MACs (G)	Peak RAM (MB)	Inference latency (ms)
ATCNet (Altaheri et al., 2022)	1.92	0.41	210 ± 10	71 ± 3
WST-CNN (Buragohain et al., 2024)	2.21	0.49	245 ± 12	89 ± 4
DB-ATCNet (Zhou et al., 2024)	2.83	0.62	290 ± 15	105 ± 5
MAML-LNN (Raza and Yusoff, 2026)	0.82	0.17	98 ± 4	26 ± 1
**Proposed 1D-CNN**	**0.120**	**0.08383**	**32.58 ± 0.655**	**46.702** **±0.448**

Bold values indicate the results obtained by the proposed method and Percentage Accuracy of proposed work.

## 5 Conclusion

In this work, we developed a framework for integrating the sliding window, Fourier decomposition method and a 1D-CNN for the interpretation of MI activities, such as right hand, left hand, both feet, and tongue. Through extensive experimental evaluation, our approach yielded compelling results on public datasets such as BCIC IV 2a and BCIC III IVa. The proposed model attained the highest classification accuracies of 79.14 ± 12.82% and 93.67 ± 9.91% in the subject-dependent multiclass and binary classifications, respectively. Furthermore, under the subject-independent LOSO evaluation protocol, the proposed framework attained average classification accuracies of 55.50 ± 19.01% on the BCIC IV 2a dataset and 76.49 ± 14.74% on the BCIC III IVa dataset. Despite the encouraging results, several limitations of the proposed framework should be acknowledged. First, cross-subject generalization remains challenging, as reflected by the relatively large inter-subject variability observed under the LOSO evaluation, particularly for the four-class BCIC IV-2a dataset. In addition, the adopted subject-independent evaluation is calibration-based, since 20% of the held-out target subject's trials were used for model adaptation; therefore, the reported performance does not represent strict zero-shot generalization to completely unseen subjects. Second, the framework was evaluated on only two public MI-EEG datasets, and cross-dataset generalization was not investigated. Finally, the Jetson Nano evaluation was performed using prerecorded EEG data and therefore does not represent complete real-time online BCI operation. Future work will focus on larger and more diverse subject populations, strict zero-shot and cross-dataset evaluation and real-time online validation using continuous EEG acquisition and decision making. Deployment on the NVIDIA Jetson Nano further demonstrated the computational efficiency of the proposed framework. The proposed 1D-CNN achieved pure inference latencies of 7.155 ms/window and 1.864 ms/window for the BCIC IV 2a and BCIC III IVa datasets, respectively, while the complete offline processing pipeline achieved latencies of complete pipeline latencies of 58.119 ms/trial and 85.055 ms/trial, corresponding to throughputs of 17.21 and 11.76 trials/s, respectively. These results highlight the deployment potential of the developed framework for low-power embedded EEG-based BCI systems. Future research could involve developing distinct MI datasets under varying experimental setups to evaluate the developed model, integrating DL models such as Recurrent Neural Networks and transformers to enhance accuracy, and deploying the model on edge platforms, including field-programmable gate arrays to further evaluate computational efficiency and hardware implementation feasibility for BCI applications.

## Data Availability

The original contributions presented in the study are included in the article/supplementary material, further inquiries can be directed to the corresponding author.
